# β-Arrestin2 promotes docetaxel resistance of castration-resistant prostate cancer via promoting hnRNP A1-mediated PKM2 alternative splicing

**DOI:** 10.1007/s12672-023-00740-0

**Published:** 2023-11-29

**Authors:** Yuhao Zhou, Fei Li, Bangyu Zou, Xiaofeng Zhou, Lianmin Luo, Sicheng Dong, Zhiqing He, Zhixiong Zhang, Liqiong Liao, Hongxing Liu, Chao Cai, Di Gu, Xiaolu Duan

**Affiliations:** 1 Department of Urology, Minimally Invasive Surgery Center, The First Affiliated Hospital of Guangzhou Medical University, Guangdong Key Laboratory of Urology, Guangzhou Institute of Urology, Kangda Road 1, Haizhu District, Guangzhou, 510230 Guangdong China; 2 Department of Pharmacy, The Fifth Affiliated Hospital of Sun Yat-sen University, Zhuhai, China

**Keywords:** β-Arrestin2, PKM2, hnRNP A1, Docetaxel resistance, CRPC

## Abstract

**Purpose:**

To investigate the influence of β-arrestin2 on the docetaxel resistance in castration-resistant prostate cancer (CRPC) and elucidate the underlying molecular mechanisms.

**Methods:**

PC3 and DU145 cells with stable β-arrestin2 overexpression and C4-2 cells with stable β-arrestin2 knockdown, were constructed via using lentivirus and puromycin selection. MTT and colony formation assays were carried out to investigate the effect of β-arrestin2 expression on the docetaxel resistance of CRPC cells. Glycolysis analysis was used to assess the glycolytic capacity modulated by β-arrestin2. GO enrichment analysis, gene set enrichment analysis and Spearman correlation test were carried out to explore the potential biological function and mechanism via using public data from GEO and TCGA. The expressions of PKM2, Phospho-PKM2, Phospho-ERK1/2 and hnRNP A1 were detected by western blot. Functional blocking experiments were carried out to confirm the roles of PKM2 and hnRNP A1 in the regulation of β-arrestin2’s biological functions via silencing PKM2 or hnRNP A1 expression in cells with stable β-arrestin2 overexpression. Finally, nude mice xenograft models were established to confirm the experimental results of cell experiments.

**Results:**

β-Arrestin2 significantly decreased the sensitivity of CRPC cells to docetaxel stimulation, through enhancing the phosphorylation and expression of PKM2. Additionally, β-arrestin2 increased PKM2 phosphorylation via the ERK1/2 signaling pathway and induced PKM2 expression in a post-transcriptional manner through an hnRNP A1-dependent PKM alternative splicing mechanism, rather than by inhibiting its ubiquitination degradation.

**Conclusion:**

Our findings indicate that the β-arrestin2/hnRNP A1/PKM2 pathway could be a promising target for treating docetaxel-resistant CRPC.

## Introduction

Prostate cancer (PCa) is a leading cause of death in men globally. After androgen-ablation therapy, most advanced PCa patients will progress to castration-resistant prostate cancer (CRPC), despite initial disease regression within 2–3 years [1]. Docetaxel (DTX) has been the mainstay of chemotherapy for PCa and is the first drug to improve overall survival in CRPC patients. However, most patients treated with DTX eventually resist the drug, leading to refractory disease and disease recrudescence [2–4]. Therefore, it remains a significant challenge to identify the underlying mechanisms of DTX resistance and improve clinical outcomes for patients with CRPC.β-arrestin2 is a negative regulator of G-protein-coupled receptor (GPCR) signaling and plays an important role in cancer progression by serving as an adaptor to guide distinct pathway signals [5, 6]. Our previous study demonstrated that β-arrestin2 (also known as ARRB2) could promote CRPC cell viability and proliferation by down-regulating FOXO1, critical in modulating multiple cancer chemotherapy resistance [7–10]. β-Arrestin2 has been demonstrated to be associated with drug resistance in various cancers, including breast cancer, bladder cancer, non-small-cell lung cancer and CRPC [11–14]. However, the role of β-arrestin2 in the regulation of DTX resistance in CRPC remains unclear.

Pyruvate kinase isoenzyme M2 (PKM2) is a critical enzyme that regulates aerobic glycolysis in tumor metabolism. Accumulating evidence suggests that high expression of PKM2 is associated with poor prognosis and chemo-resistance in various cancers [15]. In estrogen receptor-positive breast cancer, PKM2 enhance adriamycin resistance by promoting aerobic glycolysis [16]. In lung cancer, silencing PKM2 reduces ATP synthesis and leads to intracellular accumulation of docetaxel, resulting in increased sensitivity to chemotherapy [17]. Additionally, inhibition of PKM2 markedly reduces chemo-resistance of advanced bladder cancer to cisplatin [18]. In PCa, PKM2 plays a role in modulating the sensitivity of cancer cells to enzalutamide and cisplatin treatments [13]. However, the specific involvement of PKM2 in regulating docetaxel resistance in CRPC has not been fully elucidated.

In this study, we investigated the impact of β-arrestin2 expression on the DTX sensitivity of CRPC cell lines and analyzed the involvement of PKM2 in β-arrestin2-mediated DTX resistance. We also investigated the underlying mechanism of PKM2 phosphorylation and expression modulated by β-arrestin2.

## Method

### Cell culture and drugs

All CRPC cell lines, including DU145, PC3 and C4-2, were purchased from ATCC (USA) and cultured as ATCC’s suggestions. PC3 and DU145 monoclonal cells expressing GV492-β-arrestin2 (LV-ARRB2) or empty vector GV492 (LV-C) were generated and cultured in the presence of puromycin (2 μg/mL). The proteasome inhibitor MG132 and the ERK1/2 inhibitor U0126 were purchased from Selleck Chemicals.

### Lentivirus packaging and cell transfection

Short hairpin RNA (shRNA) was synthesized by GenePharma (Suzhou, China). The shRNA sequences targeting human β-arrestin2, PKM2 and hnRNP A1 were 5′-GGACCGCAAAGTGTTTGTG-3′ (shRNA-ARRB2), 5′-GGAAAGAACAUCAAGAUUATT-3′ (shRNA-PKM2) and 5′-GUAUCCAUUAUCAUGUGUA (shRNA-hnRNP A1) respectively. The sequence of unrelated shRNA was 5-TTCTCCGAACGTGTCACGT-3′ (shRNA-NC). The lentivirus expression plasmids containing β-arrestin2 was constructed by Genechem (Shanghai, China) using GV492 vector. Cells were seeded in 60-mm dishes for transient transfections and transfected at 70% confluence. According to the manufacturer's instructions, the transfections were conducted with shRNAs using lentivirus soup in the presence of 1 µg/mL polybrene.

### Cell viability assay

The cell viability was detected using MTT and colony formation methods. For MTT assay, equal amounts of cells contained 0.5% FBS, with or without DTX for indicated times, at the end of the experiment, 10 μL of MTT (5 mg/mL in PBS) were added and the cells were incubated for 3 h in the humidified incubator that contained 5% CO_2_ at 37 ˚C. Then remove the medium and add 100 µL of DMSO into each well, wrap the plate in foil and shake on an orbital shaker for 10 min. The absorbance was obtained via using a microplate reader at 490 nm. For the colony formation assay, cells were seeded in 60 mm cell culture dishes at a density of 1 × 10^3^ per well and grown in complete medium with or without DTX (5 nM) for 14 days, then the cells were fixed with 4% paraformaldehyde, stained with crystal violet and the clone number was counted.

### Western blot analysis

Western blot analysis was performed following the previously described protocol [7]. In brief, cells were lysed in RIPA buffer and equal amounts of protein were loaded onto a 10% SDS polyacrylamide gel. Subsequently, the proteins were transferred to a nitrocellulose membrane and immunoblotted with antibodies. The primary antibodies used included antibodies against β-arrestin2, phospho-PKM2, PKM2, phospho-ERK1/2, ERK1/2, hnRNP A1 (Cell Signaling Technology), Ubiquitin and GAPDH (Santa Cruz). The secondary antibodies were anti-rabbit IgG conjugated with IRDye680 and anti-mouse IgG conjugated with IRDye800 (Li-COR Biosciences). The Odyssey^®^ Infrared Imaging System (LI-COR Biosciences) was used to detect proteins of interest. The band intensities were quantified with respect to GAPDH using ImageJ software.

### Quantitative real-time polymerase chain reaction (RT-qPCR) analysis

RNA extraction from prostate cancer cells was performed using TRIzol reagent (Cat# 15596026, Invitrogen) following the manufacturer's instructions. Subsequently, 1 μg of RNA was reverse transcribed into cDNA using the PrimeScript RT Reagent Kit (Cat #RR047A, TaKaRa, Japan). The RT-PCR analysis was conducted on a LightCycler 480 System (Roche, Basel, Switzerland) using TB green (Cat #RR420A, TaKaRa) as the fluorescent dye. Specific primers for each target gene were utilized, and their sequences are shown in Table 1. The expression levels of the target markers were normalized to the expression of GAPDH.

**Table 1:** List of primers used in this study

Gene	Forward (5'-3')	Reverse (5'-3')
GAPDH	GGACCTGACCTGCCGTCTAG	GTAGCCCAGGATGCCCTTGA
PKM	ATGTCGAAGCCCCTAGTGAA	TGGGTGGTGAATCAATGTCCA
PKM1	CCACTTGCAGCTATTCGAGG	CTGCAGCACTTGAAGGAGG
PKM2	CTATCCTCTGGAGGCTGTGC	GTGGGGTCGCTGGTAATG
hnRNPA1	GCCCAGTCCATCACAATCAC	GATGCTGGCCGAGTAGGAG

### Microarray data

The matrix files of the gene expression profile of GSE33455 and GSE158494 datasets were extracted from the Gene Expression Omnibus (GEO) database (https://www.ncbi.nlm.nih.gov/geo/). Both of them contain the expression data from docetaxel-resistant prostate cancer cell lines. Batch effects were removed through the “Rtsne” package (version: 0.15). The differential expression of β-arrestin2 is analyzed by t-tests.

### GO enrichment analysis

GO enrichment analysis using the different expression genes were performed with R language with the aid of packages clusterProfiler, enrichplot and ggplot2. Only terms with both p- and q-value < 0.05 were considered significantly enriched.

### Gene set enrichment analysis

The reference gene set “c5.bp.v7.1.symbols.gmt” were downloaded from Molecular Signatures Database as the target sets with which GSEA performed using the software GSEA_4.3.2 (http://software.broadinstitute.org/gsea/index.jsp). The threshold was set at |Normalized Enrichment Score (NES)|> 1 and P < 0.01.

### Glycolysis analysis

Glycolysis analysis was carried out as previously described [19]. A Seahorse XF Cell Mitostress test kit and XFe24 extracellular flux analyzer (Seahorse Bioscience) were used to measure extracellular acidification rate (ECAR). In brief, 1 × 10^5^ cells were seeded into 24-well plates and incubated for 24 h. After the cells were washed with Seahorse buffer, glucose, oligomycin, and 2-deoxy-glucose (2-DG) were added to measure the ECAR. The ECAR values were calculated after normalization to cell number and plotted as the mean ± SD.

### *Correlation* test

Public data of β-arrestin2, PKM2, hnRNP A1, hnRNP A2B1 and PTP1B expressions in PCa patients were obtained from The Cancer Genome Atlas Project (TCGA). After excluding patients with incomplete information or normal types, 498 PCa patients’ expression data were analyzed.

### Immunoprecipitation

Immunoprecipitation was conducted as we described previously [7]. In brief, cells were mechanistically broken in ice-cold RIPA buffer and incubated with indicated antibody or control IgG antibody (Santa Cruz) at 4 °C overnight. Then the lysate mixture was centrifuged at 4000 rpm/min for 5 min and washed. The precipitated proteins were eluted for western blot analyses.

### Nude mice xenograft experiment

The nude mice xenograft experiment was carried out [20]. The male nude mice were purchased from the Experimental Animal Center of Guangdong province (Guangzhou, China). A total of 100 μL cells (1 × 10^7^ cells/mL) were subcutaneously injected into the axillary region of mouse. Tumor size was measured twice a week with a vernier caliper. Tumor volume was calculated by the formula 0.524 × (length) × (width)^2^. Mice were injected docetaxel (5 mg/kg) intraperitoneally once a week when tumor volume was approximately 100 mm^3^. After 4 weeks observed, the mice were sacrificed and the tumors were dissected, weighted and immunohistochemical stained.

### Immunohistochemical (IHC) staining

Immunohistochemistry was performed as we previous described [20]. The tissue samples were fixed, paraffin-embedded, sectioned at 4-μm thickness and then stained according to standard IHC protocol. Images were obtained via using a PathScope^™^ 4S scanner (DigiPath, USA) and quantified using Image Pro Plus software.

### Statistical analysis

The data are reported as the means ± SD of at least three independent experiments. The mean differences were compared using ANOVA and the Student t-test. A P value of less than 0.05 was considered to be statistically significant.

## Results

### β-Arrestin2 induces the chemo-resistance of CRPC cells to DTX

**Fig. 1:**
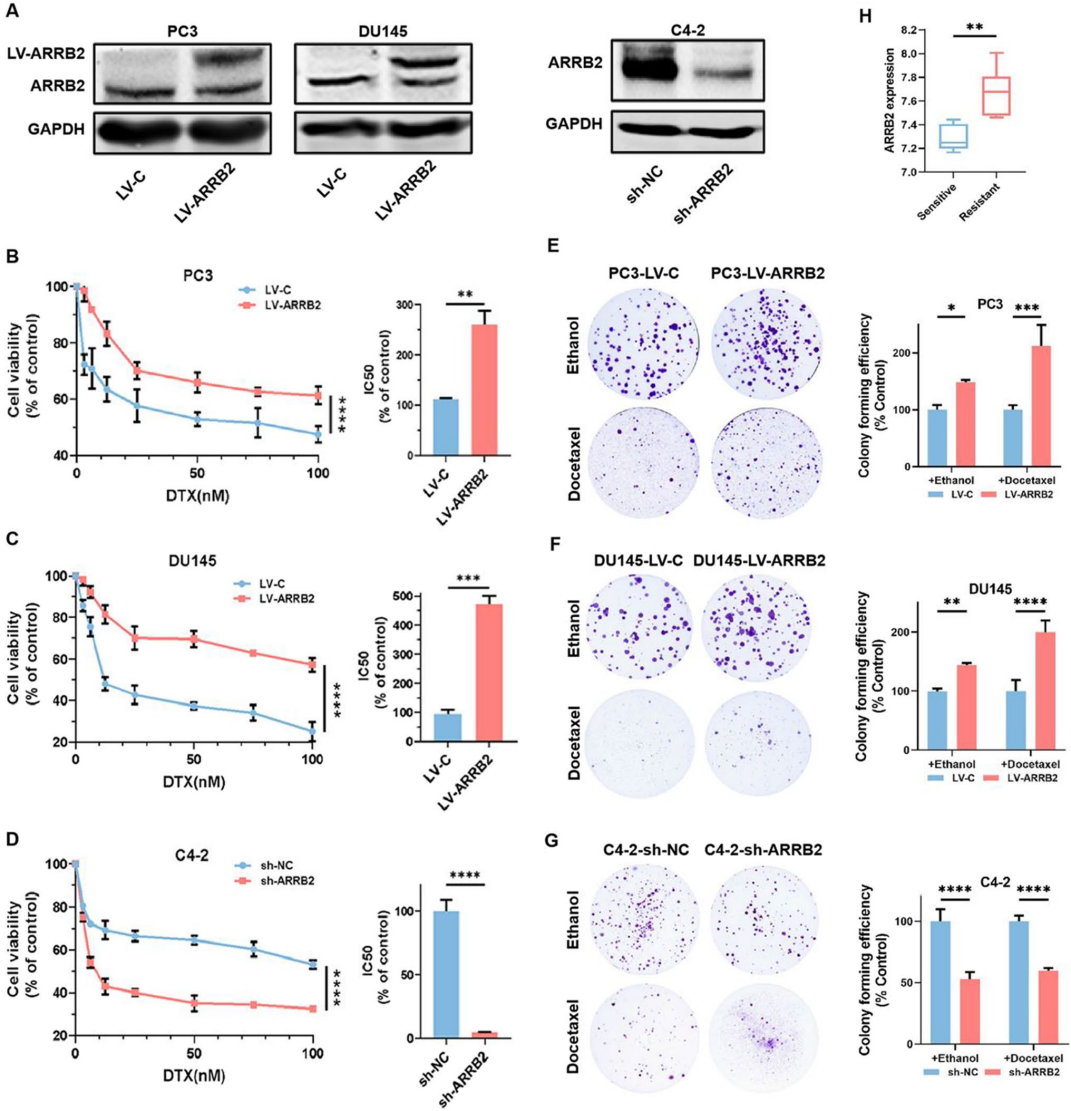
β-Arrestin2 induces the chemo-resistance of CRPC cells to DTX. **A** The expressions of the indicated proteins in PC3, DU145 and C4-2 monoclonal cells with stable ARRB2 overexpression or knockdown were detected by western blot. **B**–**D** Cell growth assay was performed by MTT and the results were expressed by the percentage of viable cells. Cells were treated with different concentrations of DTX (0–100 nM) for 48 h followed by MTT assay. **E**–**G** Colonic formation assays were used to test the cell viability of distinct cell lines cultured with ethanol or DTX (5 nM) for 14 days, then the cells were fixed and stained. **H** Merging GSE33455 and GSE158494 datasets and corrected for batch effects. Compare the expression of ARRB2 in DTX-resistance and DTX-sensitive prostate cancer cells. *P < 0.05; **P < 0.01; ***P < 0.001; ****P < 0.0001. ARRB2: β-arrestin2. C: control; NC: negative control; DTX: docetaxel

Firstly, we used PC3 and DU145 monoclonal cells with β-arrestin2 overexpression (LV-ARRB2), as well as C4-2 monoclonal cells with β-arrestin2 knockdown (LV-shARRB2), to investigate the involvement of β-arrestin2 in docetaxel (DTX) resistance (Fig. 1A). MTT and colony formation assays were performed to measure cell viability under various concentrations of DTX in parental and β-arrestin2 overexpression (or knockdown) cells. As shown in Fig. 1B–D, the IC50 values of PC3 and DU145 cells overexpressing β-arrestin2 were significantly higher than those of parental cells, whereas the IC50 value of C4-2 cells with β-arrestin2 knockdown was significantly lower than that of parental cells. The colony formation assays exhibited similar results to MTT assays (Fig. 1E–G). In addition, we found that β-arrestin2 expression was significantly increased in DTX-resistant cells compared to DTX-sensitive cells, as evidenced by data from public databases (Fig. 1H). These findings collectively suggest that β-arrestin2 could promote the tolerance of CRPC cells to DTX treatment.

### PKM2 is involved in β-arrestin2-induced DTX resistance of CRPC cells

**Fig. 2:**
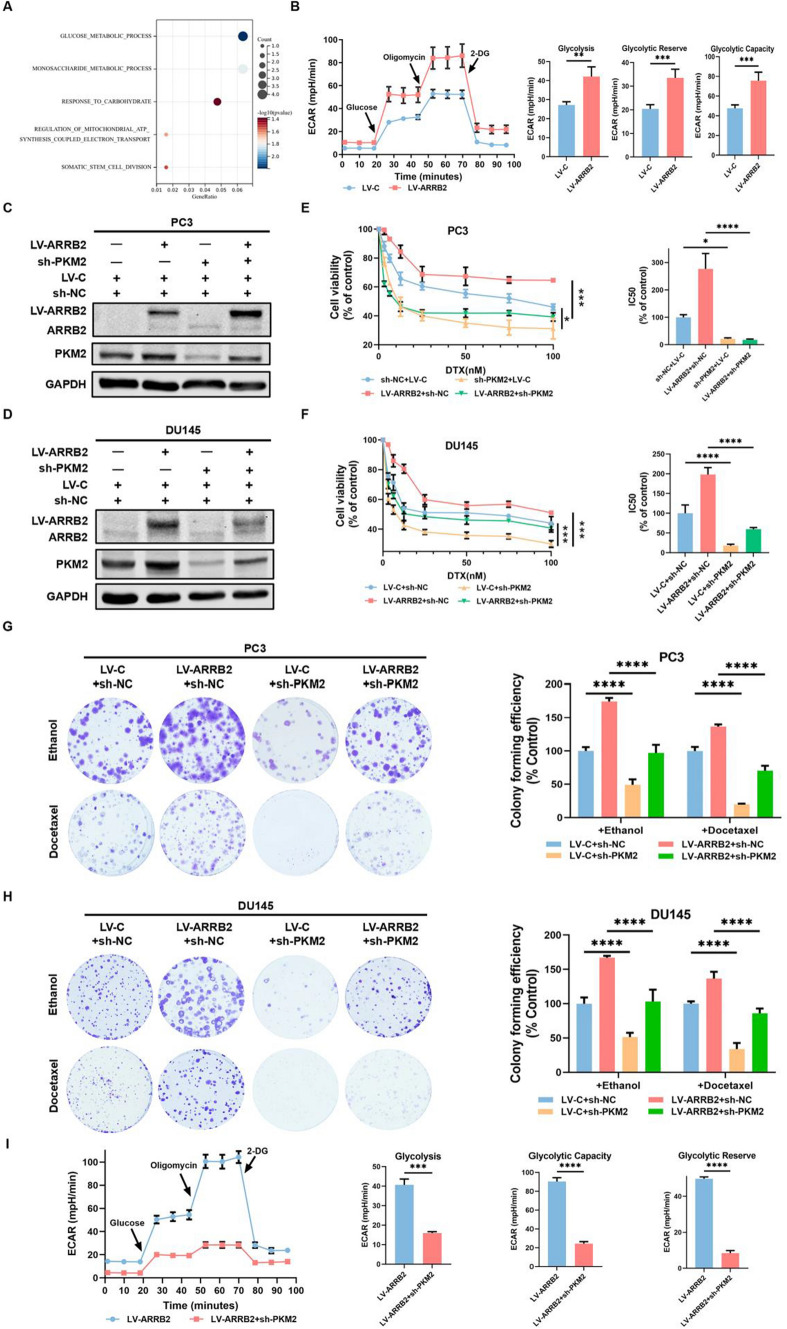
PKM2 is involved in β-arrestin2-induced DTX resistance of CRPC cells. **A** The GO analysis based on the ARRB2 and correlated genes (P < 0.05) in DTX-resistance prostate cancer. **B** A representative graph output from XFe24 showing ECAR response to glucose (10 mM), oligomycin (1 μM), and 2-DG (50 mM), and glycolytic activities were examined using the XF Analyzer. **C**–**D** The cell lines with stable LV-C or ARRB2 expression were transfected with either shRNA-PKM2 or negative control. The expressions of the indicated proteins were detected by western blot. **E**–**H** The cell viability was measured by MTT and colonic formation assays as described in Fig. 1. **I** The effect of PKM2 on ARRB2-induced glycolytic activity was determined by XF Analyzer. *P < 0.05; **P < 0.01; ***P < 0.001; ****P < 0.0001

To explore the underlying mechanisms involved in β-arrestin2-induced DTX resistance, we performed GO analysis using public database. The analysis revealed that several biological processes, including glucose metabolic, monosaccharide metabolic, response to carbohydrate, regulation of mitochondrial ATP synthesis coupled electron transport and somatic stem cell division, were significantly altered in DTX-resistant CRPC cells with high β-arrestin2 expression compared to those with low β-arrestin2 expression, separated by the median value (Fig. 2A). To investigate the effect of β-arrestin2 on CRPC metabolism, we detected the effect of β-arrestin2 on extracellular acidification rate (ECAR) in PC3 cells and observed that β-arrestin2 overexpression significantly increased the steady state glycolysis flux, glycolytic capacity and glycolytic reserve, indicating that β-arrestin2 enhances the glycolytic rate and aerobic glycolysis in CRPC cells (Fig. 2B). Since PKM2 is a critical enzyme in aerobic glycolysis and tumor metabolism, we then investigated the role of PKM2 in the regulation of β-arrestin2-induced DTX resistance in CRPC cells. As shown in Fig. 2C–H, PKM2 knockdown obviously attenuated β-arrestin2-induced cell viability and colony formation to DTX treatment in PC3 and DU145 cells. Moreover, PKM2 knockdown significantly reduced β-arrestin2-mediated increase in ECAR. Taken together, these results indicate that PKM2 is involved in β-arrestin2-induced DTX resistance of CRPC cells.

### β-Arrestin2 increased PKM2 phosphorylation and expression

**Fig. 3:**
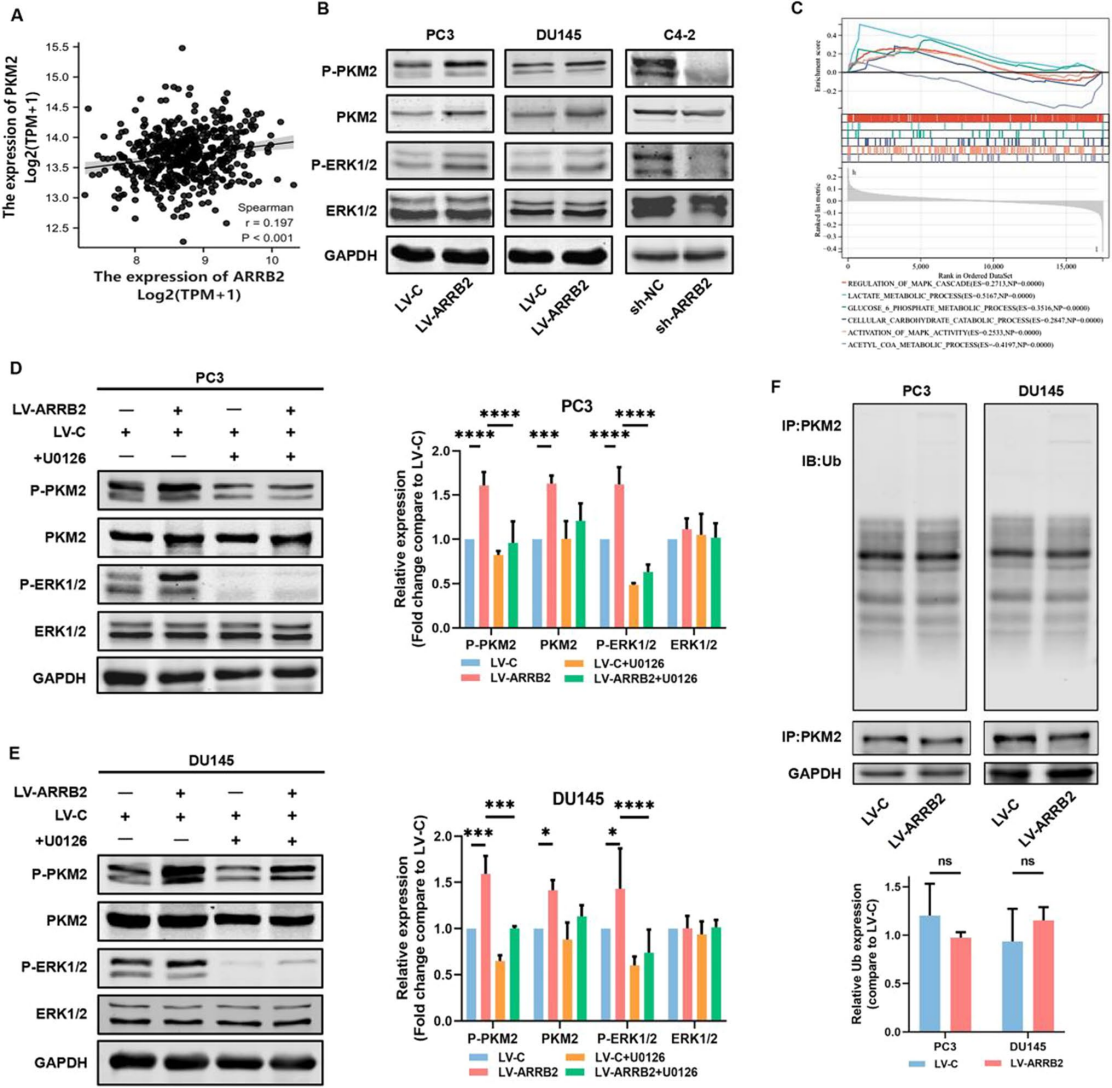
β-Arrestin2 increased PKM2 phosphorylation and expression. **A** The correlation between ARRB2 and PKM2 expression in human prostate cancer tissues was analyzed via using the Spearman’s correlation test. **B** The expressions of indicated proteins in distinct prostate cancer cell lines were detected by western blot. **C** The GSEA enrichment analysis of ARRB2 in docetaxel resistant prostate cancer cells. **D**–**E** Twenty-four hours after seeded, the distinct cell lines were treated with or without U0126 (10 μmol/L) for 1 h, then the expressions of indicated proteins were detected by western blot and quantified with respect to GAPDH using Image J software. **F** Twenty-four hours after seeded, the distinct cell lines were cultured with MG132 (10 μmol/L) for 6 h, then the cell lysates were subjected to immunoprecipitation with PKM2 antibody, subsequently the ubiquitination level was detected by western blot and quantified with respect to GAPDH using Image J software. NS denotes no significant difference; *P < 0.05; ***P < 0. 001; **** P < 0.0001

Subsequently, we investigated the expression correlation between β-arrestin2 and PKM2 based on the public data from TCGA database. As shown in Fig. 3A, a significant positive relationship existed between β-arrestin2 and PKM2 expressions (Spearman’s coefficient = 0.197; P  < 0.001). We then examined the effect of β-arrestin2 on PKM2 phosphorylation and expression. The results showed that β-arrestin2 overexpression significantly increased the phosphorylation and expression of PKM2 in PC3 and DU145 cells, whereas β-arrestin2 knockdown represented opposite effects in C4-2 cells (Fig. 3B). As GSAE assay result indicated a positive correlation between β-arrestin2 expression and the regulation and activity of the MAPK cascade (Fig. 3C). We also found that β-arrestin2 overexpression significantly increased ERK1/2 phosphorylation, while having no significant effect on total ERK1/2 protein expression (Fig. 3B, C). In addition, β-arrestin2-induced PKM2 phosphorylation was markedly attenuated by ERK1/2 inhibitor U0126 pretreatment, indicating that β-arrestin2-induced PKM2 phosphorylation is mediated by ERK1/2 signaling pathway (Fig. 3D, E). Although β-arrestin2 could interact with PKM2 to form a complex, it did not have a noticeable effect on PKM2 ubiquitination, suggesting that β-arrestin2 may induce PKM2 expression through a non-ubiquitination degradation mechanism. (Fig. 3F).

### hnRNP A1 mediates β-arrestin2-induced PKM2 expression

**Fig. 4:**
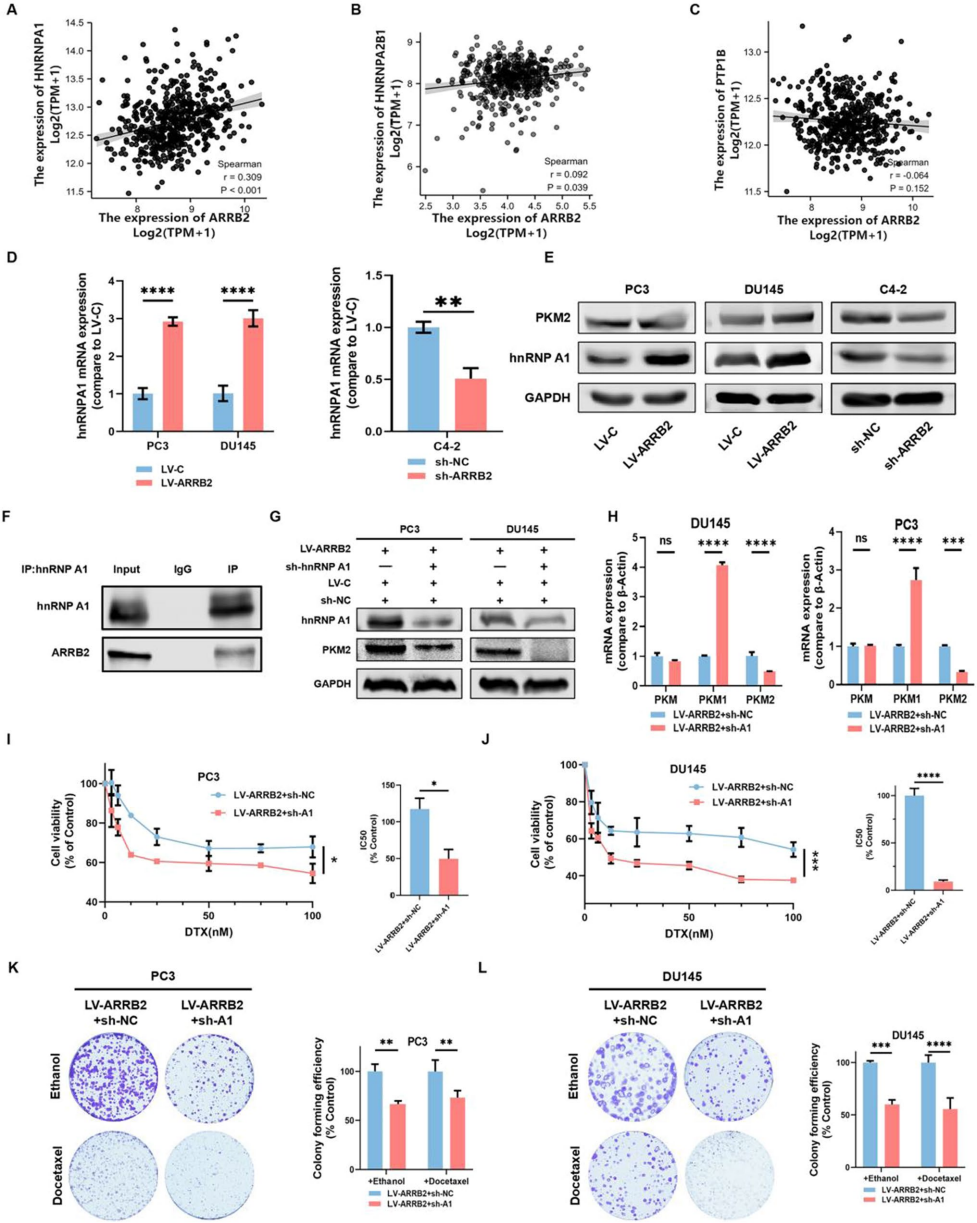
hnRNP A1 mediates β-arrestin2-induced PKM2 expression. **A**–**C** The correlation between ARRB2 and hnRNP A1, hnRNP A2B1, PTP1B expressions in human prostate cancer tissues was analyzed using the Spearman’s correlation test. **D**–**E** The expressions of indicated mRNA and proteins in distinct prostate cancer cell lines with ARRB2 overexpression or knockdown were detected using RT-qPCR and western blot. **F** The cell lysates were subjected to immunoprecipitation with hnRNPA1 or IgG antibody, subsequently the ARRB2 level was detected by western blot. **G**–**H** The expressions of the indicated proteins and mRNA in stable ARRB2-overexpressed cell lines which transfected with hnRNP A1 shRNA or a negative control shRNA were detected by western blot and RT-qPCR. **I**–**L** The cell viability was measured by MTT and colonic formation assays. *P < 0.05; ** P < 0.01; ***P < 0.001; ****P < 0.0001. A1: hnRNP A1

As PTP1B, hnRNP A1 or hnRNP A2B1-mediated PKM alternative splicing is another critical post-transcriptional mechanism for regulating of PKM2 expression [21], we analyzed the expression correlation between PTP1B, as well as hnRNP A1 and hnRNP A2B1, and β-arrestin2 based on the public data from TCGA database. As shown in Fig. 4A–C, a significant positive correlation was observed between hnRNP A1 and β-arrestin2 expressions (Spearman’s coefficient = 0.309; P < 0.001), whereas a weaker correlation relationship was found between hnRNP A2B1 and β-arrestin2 expressions (Spearman’s coefficient = 0.092; P = 0.039), and no significant relationship was found between PTP1B and β-arrestin2 expressions (Spearman’s coefficient = 0.064; P = 0.152). RT-qPCR and Western blot results also showed that β-arrestin2 overexpression notably increased hnRNP A1 expression, whereas β-arrestin2 knockdown presented the opposite effect (Fig. 4D, E). In addition, β-arrestin2 could interact with hnRNPA1 and form a complex (Fig. 4F). Furthermore, silencing hnRNP A1 expression markedly attenuated PKM2 expression but upregulated PKM1 expression in β-arrestin2 overexpression PCa cells, while having no significant effect on PKM mRNA (Fig. 4G, H). As shown in Fig. 4I–L, silencing hnRNP A1 expression markedly attenuated PKM2 expression, cell vitality and colony formation under DTX treatment in β-arrestin2 overexpressed PC3 and DU145 cells. Together, these findings suggest that β-arrestin2 may induce PKM2 alternative splicing through hnRNP A1-mediated post-transcriptional mechanism.

### β-Arrestin2 promotes tumor growth and resistance to DTX in vivo

**Fig. 5:**
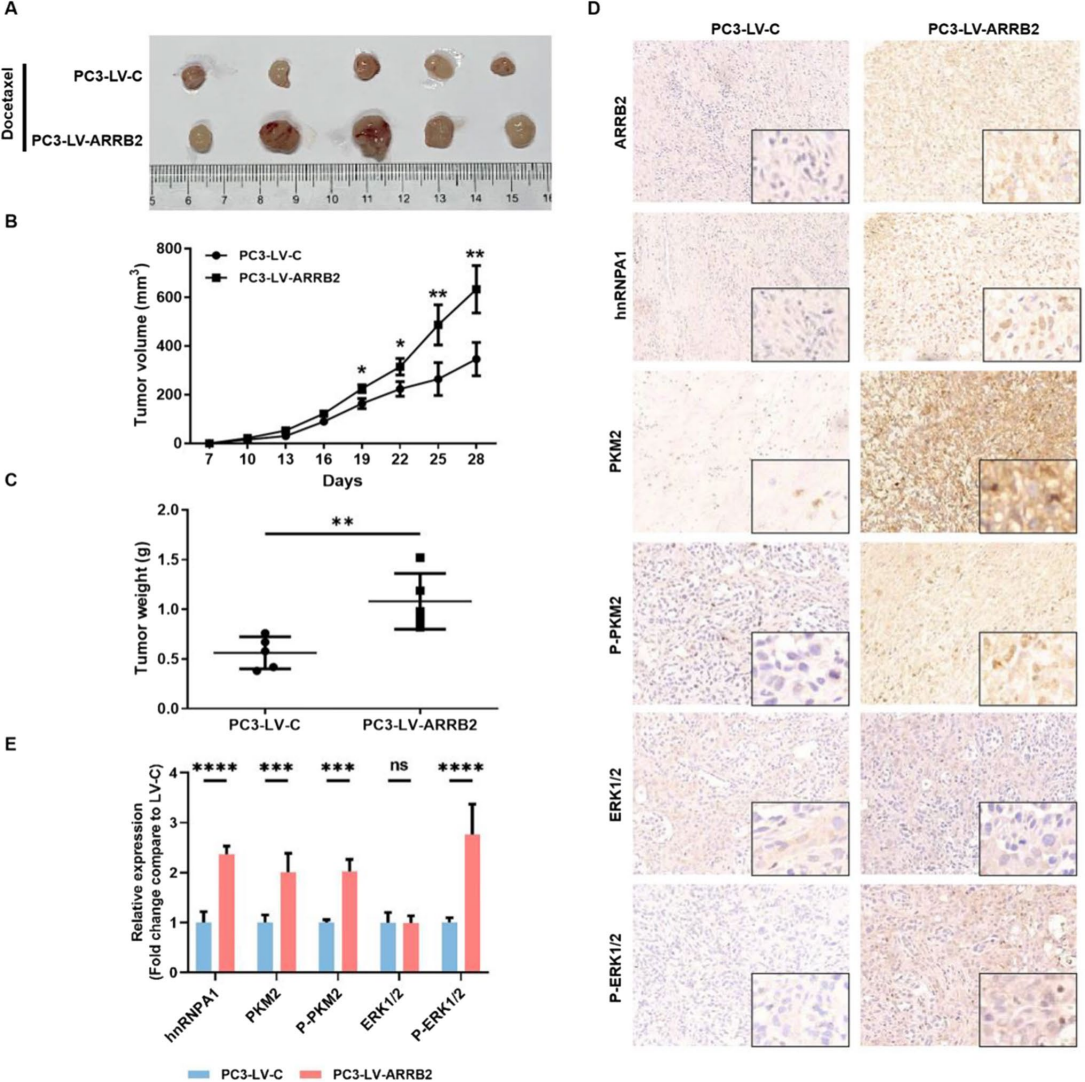
Effect of β-arrestin2 on tumor growth and resistance to DTX in vivo. **A** The representative photograph of tumors derived from nude mice injected with PC3-LV-C cells and PC3- LV-ARRB2 cells (n = 5). **B** The xenograft tumor volume of nude mice in each group (n = 5). **C** The tumor weight of nude mice in each group (n = 5). **D**–**E** Immunohistochemical staining and quantification for indicated protein in tumor samples. Original Magnification: 400×, *P < 0.05; **P < 0.01; ***P < 0.001; ****P < 0.0001

To further confirm the effects of β-arrestin2 on CRPC cell DTX tolerance in vitro, nude mice xenograft tumor model assays were conducted. As shown in Fig. 5A, the tumor volumes were significantly smaller in the mice injected with PC3-LV-C cells compare to those injected with PC3-LV-ARRB2 cells at the indicated time points, and the representative image of the tumors excised from each group was shown in Fig. 5B. In addition, the average weight of tumors in the PC3-LV-C group was markedly lower than that in PC3-LV-ARRB2 group (Fig. 5C). Furthermore, immunohistochemistry staining also revealed that the phosphorylation levels of ERK1/2 and PKM2, as well as PKM2 and hnRNP A1 expressions, in the tumors of the PC3-LV-ARRB2 group were significantly increased than that in PC3-LV-C group (Fig. 5D). Together, these results suggested that β-arrestin2 could promote CRPC progression and chemo-resistance to DTX, at least in part, via modulating ERK1/2-mediated PKM2 activation and hnRNPA1-mediated PKM2 expression.

## Discussion

This study aimed to examine the influence of β-arrestin2 on the docetaxel (DTX) tolerance in castration-resistant prostate cancer (CRPC) and uncover the underlying mechanism involved. Our findings demonstrated that β-arrestin2 was up-regulated in both DTX-tolerant CRPC cell lines and clinical samples, and that β-arrestin2 overexpression could enhance the tolerance of CRPC cells to DTX treatment both in vitro and in vivo. In contrast, β-arrestin2 knockdown represented the opposite effect. Mechanistically, we found that β-arrestin2 could promote the phosphorylation and expression of PKM2, which was essential for cell metabolism and DTX tolerance, by enhancing ERK1/2 phosphorylation and hnRNP A1 expression. Together, our findings provide novel insights into the role and underlying mechanism of β-arrestin2 in regulating DTX tolerance in CRPC.

Despite significant improvements in the treatment of prostate cancer (PCa), patients with advanced disease will ultimately progress to metastatic castration-resistant prostate cancer (mCRPC) [1, 3]. While various drugs, including docetaxel (DTX), the first-line therapy for mCRPC, initially lead to disease regression, resistance to these drugs ultimately renders mCRPC incurable [2, 4]. Therefore, understanding the mechanisms of drug resistance and identifying new therapeutic targets remain crucial in delaying or reversing the progression of CRPC.β-arrestins, including β-arrestin1 and β-arrestin2, are identified as negative regulators of G-protein-coupled receptor (GPCR) signaling in initial. Besides, subsequent research findings reveal that β-arrestins also play pivotal roles in various diseases via modulating multiple signaling pathways. In tumor, β-arrestins are involved in tumorigenesis, tumor growth, angiogenesis, tumor metabolism, invasion, metastasis and drug resistance via serving as scaffold proteins [22–24]. Recently, accumulating evidence has indicated the critical roles of β‑arrestins in PCa occurrence and progression. Our previous studies have also revealed that β‑arrestins could serve as tumor promoters by promoting cell proliferation, invasion and migration in CRPC [7, 20]. Although recent research has found that β‑arrestin2 is essential for CXCR7-mediated enzalutamide resistant in prostate cancer, little is known about the function of β‑arrestin2 in DTX-resistant CRPC [13]. Here, we examined the effect of β‑arrestin2 on DTX-treated CRPC cells and found that β-arrestin2 overexpression significantly decreased the sensitivity of CRPC cells to DTX treatment. The data from the public database also showed that β-arrestin2 expression was significantly increased in DTX-resistant CRPC cells, suggesting that β-arrestin2 could promote the resistance of CRPC to DTX treatment. Notably, the effect of β-arrestin2 on chemotherapy tolerance in CRPC is similar to its effect in breast cancer but opposite to that in bladder cancer, indicating that the exact effect of β-arrestin2 on drug tolerance is affected by cancer type [11].

To explore the underlying mechanisms, we analyzed the potential biological processes involved in DTX resistance through GO analysis based on public database date. The results revealed significant changes in metabolic processes, including glucose metabolism and regulation of mitochondrial ATP synthesis coupled electron transport, between DTX-resistant CRPC cells with high and low β-arrestin2 expression. We then examined the effects of β-arrestin2 on glycolysis and tumor metabolism through seahorse assay, and found that β-arrestin2 overexpression significantly promoted the glycolytic rate in PC3 cells, suggesting that the enhanced aerobic glycolysis may be a key reason of DTX tolerance induced by β-arrestin2 in CRPC cells.

As PKM2 is a key enzyme of aerobic glycolysis in tumor metabolism and a key down-target of PI3K/Akt and MAPK signaling pathways [15, 25, 26], whereas β-arrestin2, a pivotal adapter in PI3K/Akt and MAPK signal transduction, is also involved in the metabolic and cellular process that responses to chemical stimulus [14, 22, 27, 28]. We then detected and found that PKM2 knockdown obviously attenuated β-arrestin2-induced DTX tolerance, as well as glycolysis in PC3 cells, suggesting that β-arrestin2-induced DTX tolerance is mediated, at least in part, by PKM2. These results are consistent with previous studies that indicated the vital role of PKM2-mediated glycolysis in the regulation of CRPC chemotherapy tolerance [13, 29, 30].

In addition, although accumulated evidences indicated the vital role of PKM2-mediated glycolysis in the regulation of numerous modulators-mediated tumorigenesis, cancer progress and chemotherapy tolerance, and has uncovered PKM2 activation-related mechanisms, little is known about the related mechanism of PKM2 expression regulation [15–18, 25, 26, 31]. In the present study, our results showed that β-arrestin2 could increase PKM2 phosphorylation via ERK1/2 signaling pathway which was good consistent with previous studies [25, 32, 33]. Notably, our results indicated that β-arrestin2 could increase PKM2 expression via a hnRNP A1-mediated post-transcriptional manner, thus revealing a novel upstream mechanism of PKM2 expression modulated by β-arrestin2.

Limitedly, we have not investigated the interaction between β-arrestin2 and hnRNPA1, nor examined the effect of β-arrestin2 on hnRNPA1-mediated PKM alternative splicing. In addition, the underlying mechanism of β-arrestin2-induced hnRNP A1 expression also requires further investigation.

Collectively, in the present study, we investigated the role and underlying mechanism of β-arrestin2 in the regulation of DTX tolerance in CRPC, our data revealed that β-arrestin2 could promote the resistance of CPRC to DTX treatment via increasing PKM2 phosphorylation and expression, which was modulated by ERK1/2 signaling pathway and hnRNPA1-mediated alternative splicing respectively. We expect that our findings on β-arrestin2/hnRNP A1/PKM2 axis-mediated DTX tolerance are helpful for providing new intervention targets and treatment strategies for DTX-resistant CRPC.

## Data Availability

The datasets generated and/or analyzed during the current study are available from the corresponding author on reasonable request Data availability Statement.
